# Supplementary material for: The cellular Potts model on disordered lattices
Source: Soft Matter. 2024 Sep 10;20(42):8337–52. doi: 10.1039/d4sm00445k (PMC11404401; doi:10.1039/d4sm00445k)
Supplement: SM-020-D4SM00445K-s001 [file SM-020-D4SM00445K-s001.pdf]

Supplementary Information (SI) for Soft Matter.
This journal is © The Royal Society of Chemistry 2024

Electronic Supplementary Information: The Cellular Potts Model on Disordered Lattices

Hossein Nemati[a*], Joost de Graaf[a]

[a] *Institute for Theoretical Physics, Center for Extreme Matter and Emergent Phenomena, Utrecht University, Princetonplein 5, Utrecht 3584 CC, The Netherlands*

*Corresponding author: Hossein (H.) Nemati; h.nemati@uu.nl

S1 Coexistence

An example snapshot in Fig. S1 shows patterns of coexistence for a simulation with $\alpha = 2$, which is close to the transition point on the disordered lattice. Figure S1a reveals that within one simulation volume, there are regions with more disordered (yellow cells are abundant) and ordered (purple cells in the bottom-right quadrant) configurations. We have plotted Voronoi tessellated version of cells in Fig. S1b, for which the colors are based on the hexatic order threshold $|\psi_6| = 0.76$; above and below indicated using purple and orange, respectively. This representation better shows the ordered cluster.

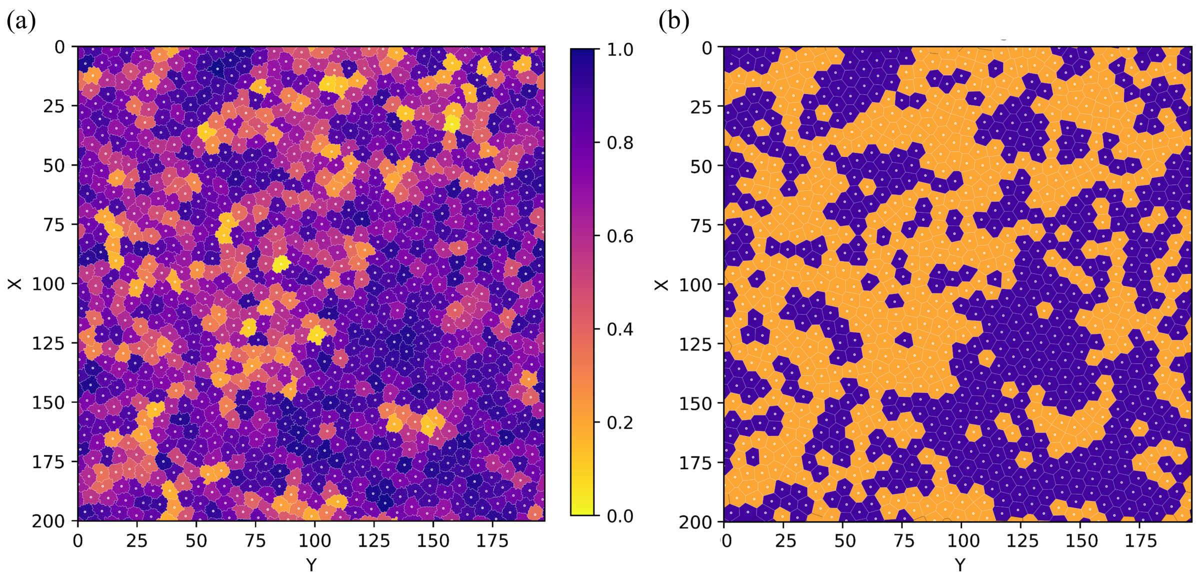

Figure S1: An example simulation snapshot revealing that an ordered and disordered phase are present in the same simulation volume for $\alpha = 2.0$. (a) Cells are colored by their local hexatic order $|\psi_6|$ bar, as indicated by the bar on the right-hand side of this panel. (b) The Voronoi-tessellated version of the same snapshot in panel (a). In this panel, the purple and orange cells have a hexatic order greater and smaller than 0.76, respectively. The colors in panel (b) are not related to the color bar used in (a). The white dots in both panels show the center of mass of the cells.

S2 Mean square displacement (MSD)

Figure S2 shows the mean-squared displacement (MSD) of the center-of-mass of cells on the disordered lattice for all values of α that were studied. They are averaged over the individual cells across the various independent simulations that were performed at a given state point. We can see that for low values of α, ($\alpha < 2.0$), the MSD is diffusive for the times shown here. For higher values of α, there is an initial plateau, which is due to caging effects. The MSD eventually becomes diffusive, though for some systems it can take a very long time. We only simulated trajectories close enough to the cross-over region between ordered and disordered for longer than 10^6 MCS.

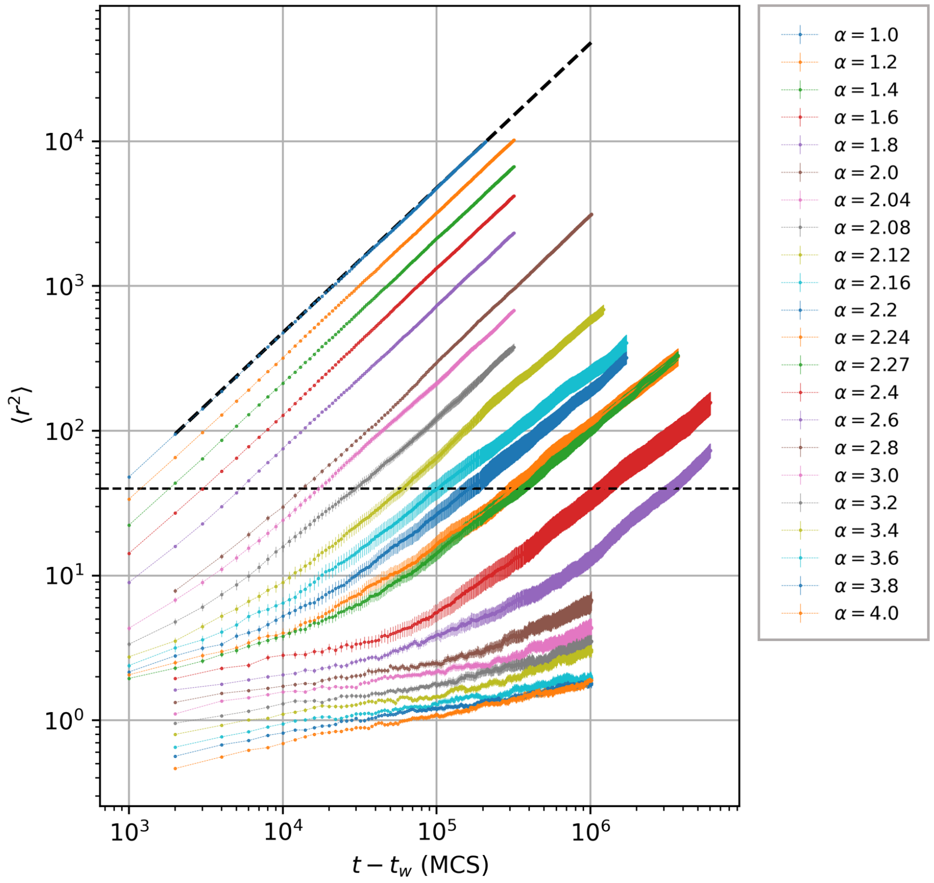

Figure S2: The average mean-squared displacements $\langle r^2 \rangle$ of the center-of-mass of cells are plotted as a function of simulation time t (starting from the waiting time t_w) for different values of α. All simulations were performed on a disordered lattice. The legend provides the values of α, but in reading the graph α increases from top to bottom. Measured points are connected to guide the eye and the error bars indicate the standard error of the mean. The horizontal dashed line indicates A_0, the target cell area. This can be used to determine the time at which a cell has displaced its own size on average. The sloped dashed line indicates a diffusive trend and partially overlaps with the $\alpha = 1.0$ data, which is thus clearly diffusive.

S3 Lattice artifacts

Figure S3 shows the probability density function (PDF) belonging to the hexatic order parameter $|\psi_6|$ as measured for a high value of $\alpha = 4$. The distribution shows a clear main peak lattice artifact for the underlying square lattice, with a potential secondary artifact distribution to the right of the vertical black line. Conversely, the distribution on the irregular lattice is smooth and shows no clear lattice artifacts.

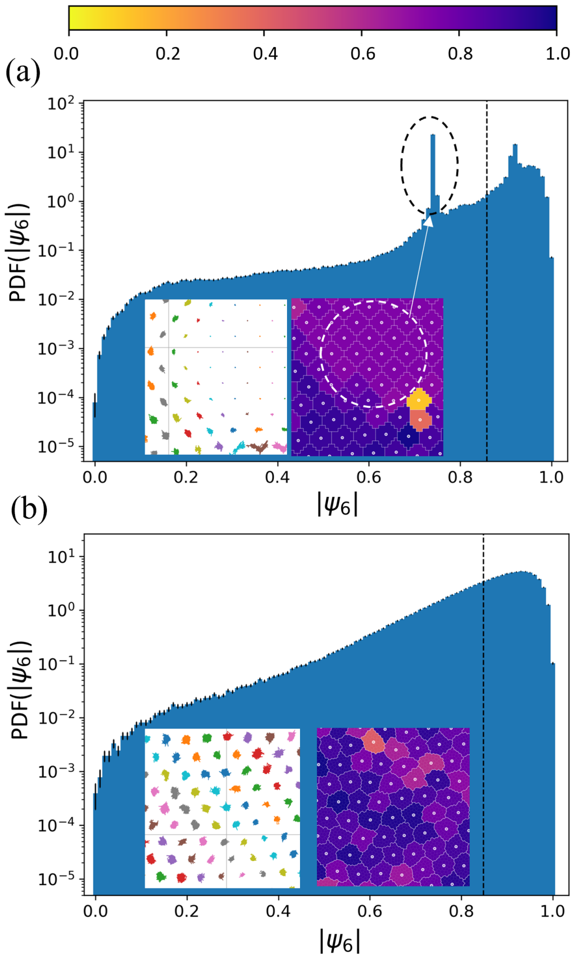

Figure S3: The lattice artifacts observed on the square lattice disappear on the disordered lattice. (a) The distribution (probability density function; PDF) of the hexatic order on the square lattice for $\alpha = 4$. The unphysical peak is indicated by the dashed ellipse. (b) The same distribution for the same value of α on the disordered lattice. The insets in both panels show the configurations of the cells and the trajectories of their centers of mass. On the right-hand side of the inset, the model cells are colored by the hexatic order parameter $|\psi_6|$, for which the color bar is shown at the top of the figure. The coloring on the left-hand side is to distinguish individual cells and has no physical significance. In both graphs, the error bars indicate the standard error of the mean and the vertical dashed line represents the average value of the distribution.

S4 Pseudo-polygons on regular lattices

Figure S4 shows the fractions of pseudo-polygons on square and hexagonal lattices. From Fig. S4a, it becomes clear that the argument we made about the crossover between f_6 and f_5 being an indicator of the phase transition on the irregular lattice, holds also for the square lattice. Figure S4b shows the fractions on the hexagonal lattice. As can be seen, on the square lattice, as with the irregular one, the transition region well matches with the crossover point between the fractions of pseudo-hexagons and pseudo-pentagons. However, for the hexagonal lattice, there is a slight mismatch. Though the mismatch is small, this could suggest that there is an anomalous diffusion behavior, caused by the underlying hexagonal order. That is, the system becomes subdiffusive, before crystallization sets in.

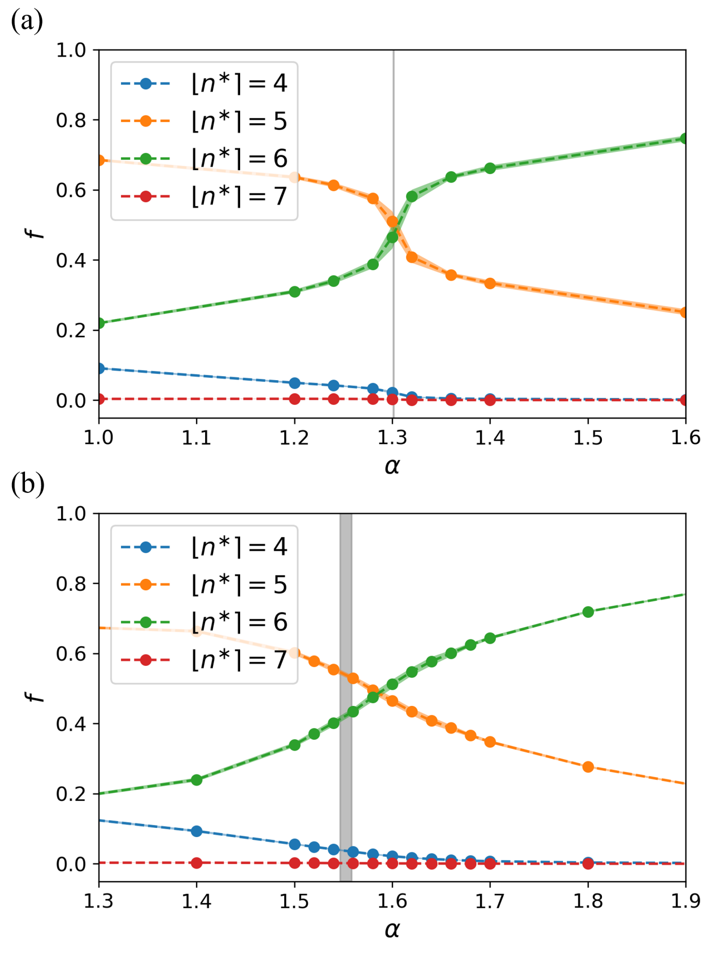

Figure S4: The fractions of pseudo-polygons f_{n*} as a function of the surface tension α obtained for square (a) and hexagonal (b) lattices. The number of edges for the pseudo-polygons is indicated in the legend and the colored area around the respective curves indicates the standard error of the mean. The vertical gray line (bar) shows where the diffusion coefficient drops, also see many text.

S5 q_V at the transition

The fractions shown in Fig. 7a in the main text derive from integrals of the q_V distributions. We can also examine the average and the median value of q_V across the transition zone. In figure S5a, we find that for the irregular and square lattices, the median of q_V being equal to $q(n^* = 5.5)$ is a very good indicator of the transition, *i.e.*, to within the standard error of the mean. However, for the hexagonal lattice, there is a mismatch, which is likely similar in origin to the one observed in Fig. S4b. Figure S5b provides the average ($\langle q_V \rangle$). Here, we clearly see that neither proposed structural measure provides an accurate location for the transition. Thus, we conclude that the median of q is the more relevant quantity.

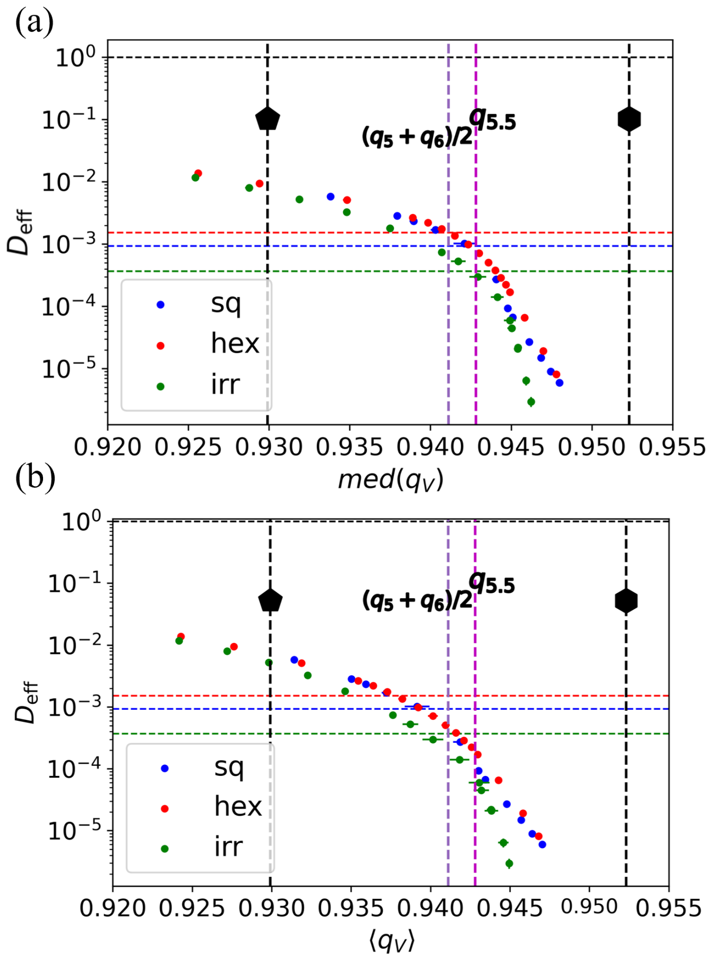

Figure S5: Behavior of the median and average of q_V across the transition. (a) The diffusion coefficient is plotted as a function of the median of q_V. (b) The diffusion coefficient as a function of the average of q_V. The three underlying lattices are as indicated using the colors in the legends and the error bars indicate the standard error of the mean. The values of q_V for a pentagon and hexagon are indicated using vertical dashed lines and symbols. The derived values for a generalized $(n^* = 5.5)$-gon and the arithmetic mean of the hexagon and pentagon values are indicated using vertical dashed magenta and purple lines, respectively. The horizontal dashed lines indicate $D_{\text{eff}}^{\blacksquare}$ on different lattices.

S6 n^* on regular lattices

Figure S6 shows the distribution of n^* parameter and the behavior of its extrema on the square and the hexagonal lattices. Like the irregular lattice, we see that the number of the extrema of this distribution determines the state. As we discussed for the irregular lattice, the local minimum and the minor local maximum merge together and disappear at the transition, while the position of the global maximum experiences an abrupt change in its slope, and hardly changes in value beyond the transition. The global maximum at the transition is around $n^* \approx 5.7$, as was the case for the irregular lattice.

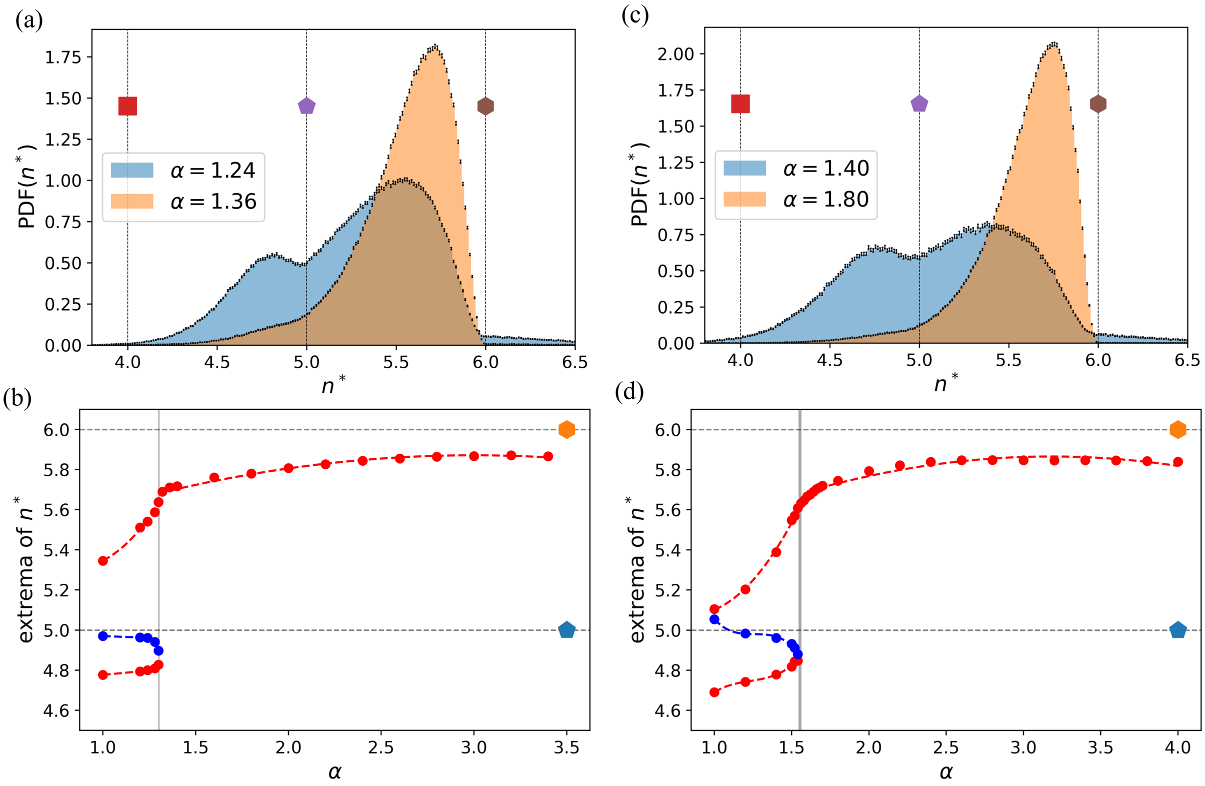

Figure S6: The distribution of n^* parameter indicates the phase transition on regular lattices, but less so on hexagonal lattices. (a) Distribution of n^* for two values of α are shown as well as the local minimum on the square lattice. For $\alpha = 1.24$ (blue) the tissue is fluid-like, while for $\alpha = 1.36$ (orange) it is solid-like. (b) The positions of the maxima (red) and the local minimum (blue) of n^* distribution on the square lattice are plotted as a function of α. The dashed lines are there to guide the eye, and the gray box indicates the transition point. (c,d) The analog of (a,b) for a hexagonal lattice, with a typical fluid-like ($\alpha = 1.4$, blue) and solid-like state ($\alpha = 1.8$, orange).

S7 Circularity on regular lattices

Figure S7 shows the behavior of the mode of the Voronoi circularity on square and hexagonal lattices. Unlike the distributions of q_V and n^*, the distribution of C_V always has only a single peak. Nonetheless, the mode of this distribution is also a complementary determinant of the transition. As for the irregular lattice we discussed in the main text, on the square and hexagonal lattices, the mode of C_V distribution is very close to the circularity of the pentagon, when the system transitions from disordered and fluid-like to ordered and solid-like.

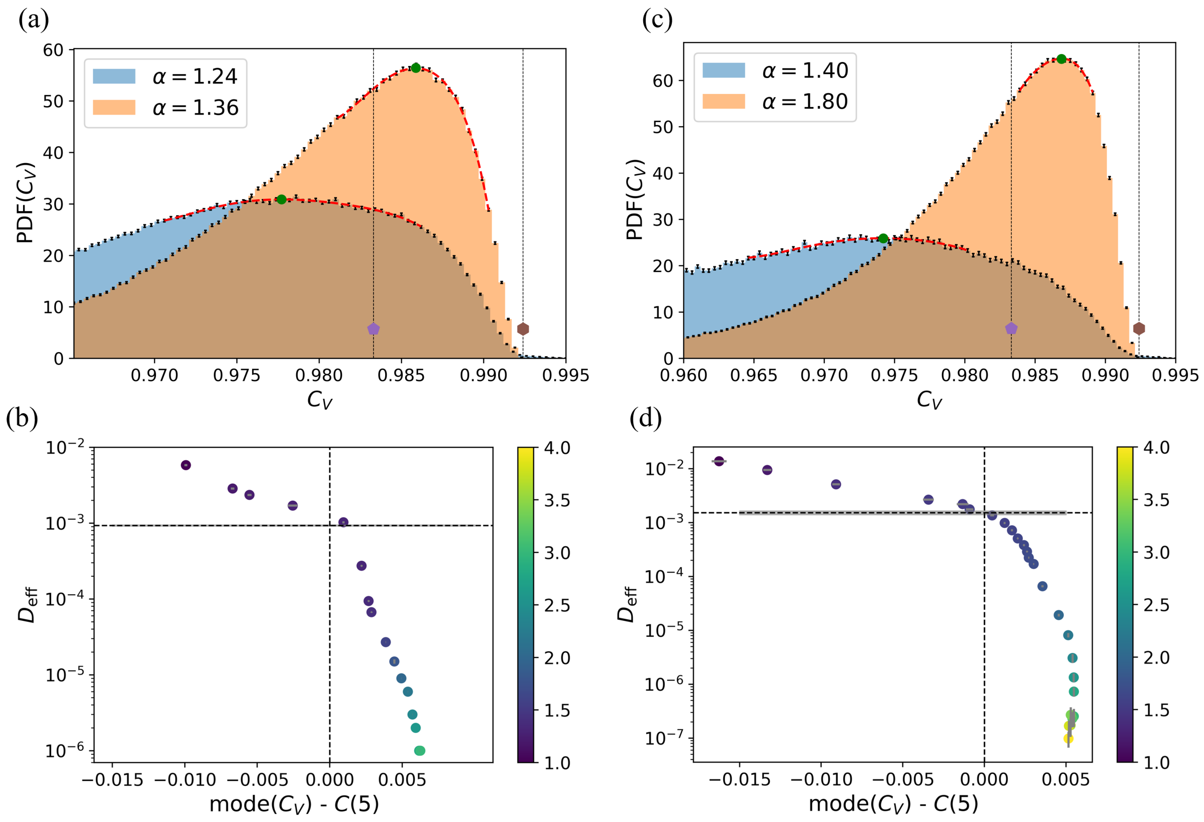

Figure S7: The mode of the Voronoi-based circularity indicates phase transition on regular lattices, (a) The PDF of the Voronoi-based circularity, C_V, for two values of α, one of which in fluid-like ($\alpha = 1.24$, blue) and the other in solid-like ($\alpha = 1.36$, orange) regime, on the square lattice. The positions of the peaks (green dots) are determined by polynomial fittings (red dashed lines). The vertical dashed lines show the values of circularity for the pentagon and hexagon. (b) The diffusion coefficient, D_{eff}, plotted as a function of the departure between the mode of C_V from the circularity of a pentagon, $C(5)$, on a square lattice. The horizontal dashed line shows the diffusion coefficient at transition, *i.e.*, $D_{\text{eff}}^{\blacksquare}$. The data points are colored by the values of α as shown by the color bar on the right. The data of the highest 3 values of α are not plotted because of the considerable lattice artifacts. (c,d) The analog of (a,b) for a hexagonal lattice, with a typical fluid-like ($\alpha = 1.4$, blue) and solid-like state ($\alpha = 1.8$, orange). The gray box in panel d indicates the standard error of $D_{\text{eff}}^{\blacksquare}$. This plotted in panel b as well, but the error is too narrow to properly visualize.
